# Monarch caterpillars are robust to combined exposure to the roadside micronutrients sodium and zinc

**DOI:** 10.1093/conphys/coab061

**Published:** 2021-08-09

**Authors:** Alexander M Shephard, Timothy S Mitchell, Emilie C Snell-Rood

**Affiliations:** Department of Ecology, Evolution, and Behavior, University of Minnesota, Twin Cities, Saint Paul, MN 55108, USA

**Keywords:** anthropogenic change sodiummicronutrientmonarch butterflynutritional ecologyzinc

## Abstract

Human activities are increasing the environmental availability of micronutrients, including sodium and some essential metals. Micronutrients are often limiting in animal diets but may have negative effects when consumed in excess. Though prior research has documented how elevated exposure to individual micronutrients can impact organismal development and fitness, we know less about combined effects of multiple micronutrients. In the wild, monarch butterfly larvae (*Danaus plexippus*) commonly consume plants in roadside habitats that contain elevated levels of sodium (from road salt) and zinc (from vehicle wear-and-tear). We reared monarch caterpillars to adulthood to test individual and combined effects of dietary sodium and zinc on components of fitness, sodium-linked phenotypes (proxies for neural and flight muscle development) and concentrations of sodium and zinc in adult butterflies. Monarch survival was not impacted by elevated sodium or zinc individually or in combination. Yet, monarchs feeding on sodium-treated milkweed developed relatively larger eyes, consistent with a positive effect of sodium on neural development. Measurements of element concentrations in butterfly and plant tissue indicated that monarchs had higher zinc levels than those present in zinc-treated milkweed but lower sodium levels than those present in sodium-treated milkweed. Monarchs developing on sodium-treated milkweed also had prolonged development time, which might be a cost associated with developing extra neural tissue or investing in mechanisms to excrete excess dietary sodium during the larval stage. Our results indicate that sodium, more than zinc, is likely influencing phenotypic development and performance of insect pollinators in roadside habitats. Yet, in contrast to previous work, our experiment suggests that the highest levels of sodium found along roads are not always harmful for developing monarchs. Future work could consider how potentially stressful effects of micronutrients could be mitigated by increased macronutrient availability or how developmental factors such as migratory status might increase micronutrient requirements.

## Teaser

Humans are increasing micronutrients in roadside habitats to levels that are potentially stressful for insect pollinators. We show that combined exposure to levels of sodium and zinc characteristic of high-traffic roadsides do not impact survival of developing monarch butterflies (*Danaus plexippus*), but sodium increases relative eye size and prolongs development.

## Introduction

Human activities are drastically altering the distribution and abundance of chemical nutrients in ecosystems (Vitousek *et al.*, 1997; Smith, 2003; Isbell *et al.*, 2013). In many cases, nutrient availability is being increased to levels beyond those historically experienced by natural populations, and this can have major effects on organismal development and fitness (Chen *et al.*, 2004; Prudic *et al.*, 2005; Snell-Rood *et al.*, 2014). Such effects are often studied in the context of major limiting macronutrients that are required by organisms in large quantities, such as nitrogen, phosphorous and carbon (Jefferies and Maron, 1997; Elser *et al.*, 2001; Galloway *et al.*, 2008; Harpole *et al.*, 2011). However, humans are also increasing the availability of many micronutrients (e.g. sodium, zinc, iron, copper and calcium), which are chemicals required by organisms only in trace amounts but have negative effects when consumed in excess (Snell-Rood *et al.*, 2015; Kaspari, 2020). For instance, sodium availability in both terrestrial and aquatic ecosystems is increasing due to crop irrigation with salt water (Rengasamy, 2006), application of road salts during winter in northern climates (Jackson and Jobbágy, 2005; Findlay and Kelly, 2011), rising sea levels and overuse of groundwater (Daliakopoulos *et al*. 2016). Sodium is a key limiting micronutrient in animals (Seastedt and Crossley, 1981; Kaspari *et al.*, 2009; Welti *et al.*, 2019; Prather *et al.,* 2020) that positively contributes to neural and muscular development (Hodgkin, 1951), but it quickly becomes toxic at higher levels (e.g. Soucek and Dickinson, 2015). Other key micronutrients are essential metals, such as zinc, iron and copper, which have crucial roles in enzyme production and development in virtually all taxa (Klaassen *et al.*, 1999; Hsieh *et al.*, 2013; Andresen *et al.,* 2018). Yet, human activities such as industrial combustion, mining and vehicle brake and tyre wear-and-tear are increasing heavy metals in ecosystems to potentially toxic levels (Councell *et al.*, 2004; Ozaki *et al.*, 2004; McKenzie *et al.*, 2009), which may negatively impact organismal performance (Nagajyoti *et al.*, 2010; Gall *et al.*, 2015).

The ecotoxicology literature contains hundreds of studies focused on stressful effects of single micronutrients on organismal development and performance (e.g. Bursey and Watson, 1983; Al-Dahhan *et al.*, 2002; Xiao *et al.*, 2010; Snell-Rood *et al.*, 2014; Shephard *et al.*, 2021). However, environmental changes in nutrient availability do not occur in isolation but rather in combination (e.g. see Kaushal *et al.*, 2020). Also, less is known about the effects of combined exposure to potentially stressful levels of multiple micronutrients. Studies of combined exposures to micronutrients may yield important conservation insights given that nutrients can interact in diverse ways to affect organismal physiology and performance (Simpson *et al.*, 2010; Kaspari and Powers, 2016). For instance, metabolic pathways consist of complex nutrient co-limitation cascades, where increases in the availability of one nutrient may increase demand for other nutrients (Sterner and Elser, 2002). Such micronutrient co-limitation can have a major influence on productivity of both terrestrial and marine ecosystems (Kaspari *et al.* 2017; Browning *et al.*, 2017). For instance, addition of micronutrients, such as sodium and zinc, can increase decomposition rates just as much as nitrogen and phosphorous additions (Powers and Salute, 2011; Kaspari *et al.*, 2014). Yet, it is less clear whether increases in the environmental availability of multiple limiting micronutrients may benefit organismal development and performance, which we refer to as the *co-limitation hypothesis*. Despite potential benefits, micronutrients may also have combined negative effects, particularly if consumed in excess, referred to as the *multiple stressors hypothesis*. For instance, combined exposures to sodium and heavy metals are known to be stressful in plants (Kholodova *et al.*, 2010; Steffens, 2014), but combined effects of these micronutrients are less studied in animals. Indeed, mechanisms in animal cells that bind and sequester heavy metals such as zinc (e.g. metallothionein proteins) may be energetically costly (Forbes and Calow, 1996), as sub-lethal metal exposures have been shown to increase organismal metabolic rates by 39–175% (Rowe 1998; Rowe *et al.*, 1998). Unlike many heavy metals, sodium cannot be stored in animal cells in a stable form (NRC, 2005), and considerable energy is therefore required to maintain cellular sodium levels by balancing intake and excretion (e.g. 1/3 of the animal cell’s resting metabolic rate is devoted to the maintenance of sodium–potassium pumps; Frausto da Silva and Williams, 2001). When faced with such high metabolic demands, it is possible that organisms may not be able to effectively regulate both internal sodium and heavy metal levels, which could result in toxicity and reduced performance (Ercal *et al*., 2001).

To advance our understanding of how anthropogenic increases in micronutrients are affecting natural populations, we need more case studies assessing how organisms respond to specific combinations of micronutrients commonly encountered in their habitats. In this research, we focus on combined effects of micronutrients in roadside habitats. Roadways are significant sources of micronutrient release, but surrounding roadside verges provide habitat for insect pollinators such as butterflies, moths and bees (Vermeulen and Opdam, 1995; Ries *et al.*, 2001; Saarinen *et al.*, 2005; Valtonen *et al.*, 2007; Hopwood, 2008; McCleery *et al.*, 2015). For instance, heavy metals such as zinc, copper and iron are commonly released from vehicle wear-and-tear and accumulate in roadside soils (Fergusson *et al.*, 1980; Ho and Tai, 1988; Jaradat and Momani, 1999; Councell *et al.*, 2004; McKenzie *et al.*, 2009). Additionally, sodium-containing salts are applied in large quantities to roads during winter for de-icing purposes (Novotny *et al.*, 2009; Kaspari *et al.*, 2010), but sodium remains in roadside soils throughout the growing season (Mitchell *et al.*, 2020). These micronutrients can contaminate leaf tissue of plants that are consumed by leaf-feeding insect pollinators such as larval butterflies and moths. Micronutrient contamination of leaf tissue along roadsides can occur either through direct uptake of chemicals by plant roots (Verbruggen *et al.*, 2009) or through accumulation of chemical dust on leaf surfaces (Shakour and Nasralla 1986). As roadside habitats are currently being targeted for restoration of pollinator-friendly plants (Phillips *et al.*, 2020), understanding the combined effects of these roadside elevated micronutrients on organismal development and performance is highly relevant to insect pollinator conservation.

In this experiment, we test the combined effects of exposure to elevated levels of sodium and zinc in developing monarch butterflies (*D. plexippus*). The charismatic monarch is a flagship insect species of conservation concern (Guiney and Oberhauser, 2009) that is currently waitlisted by the US Fish and Wildlife Service for being listed on the Endangered Species Act (Center for Biological Diversity *et al*., 2014). Roadsides have high potential for monarch conservation because they can provide high densities of larval host plants and adult nectar plants (Kasten *et al.* 2016; Thogmartin *et al.*, 2017); however, there may be risks associated with the consumption of traffic pollutants. We focus specifically on sodium and zinc because these are among the most prominent human-released micronutrients in local roadside habitats contaminating common milkweed (*Asclepias syriaca*), the most common roadside larval dietary host plant of the monarch throughout the American Midwest (Kasten *et al.*, 2016). Surveys of common milkweed collected from roadside sites across MN, USA, have shown that leaf zinc and sodium concentrations are higher in plants along busier roads and in plants closer to the road edge (Mitchell *et al.*, 2020; Shephard *et al*., in review). Prior experimental work has investigated the effects of increased dietary sodium (Snell-Rood *et al.*, 2014) and increased dietary zinc (Shephard *et al.*, 2020) on monarch development in isolation, but not yet in combination. Increased dietary sodium enhances neural and muscle development in monarchs, but can reduce survival at high levels (Snell-Rood *et al.*, 2014). Despite elevated levels of sodium in roadside milkweed (Mitchell *et al.*, 2020) neither caterpillars nor ovipositing females avoid leaves with toxic sodium levels (Mitchell *et al.*, 2019). Roadside milkweed accumulates sodium to levels that are potentially toxic for monarchs, but observed levels of zinc in roadside milkweed are likely not toxic for monarchs (Mitchell *et al.*, 2020; Shephard *et al.*, 2020). It remains unclear how elevated zinc affects monarch development and performance when experienced in combination with other micronutrients, such as sodium.

We captured monarch butterflies from wild populations in MN, USA, and reared cohorts of their offspring on milkweed dosed with elevated levels of sodium and zinc in a 2 × 2 factorial experiment. Our first aim was to determine the combined effects of elevated sodium and zinc on monarch survival and investment in life history traits and we considered two possible hypotheses. The multiple stressor hypothesis predicts that given the potentially high energetic demands of coping with elevated dietary micronutrients (Frausto da Silva and Williams 2001; Rowe 1998; Rowe *et al.* 1998), monarchs will not be able to effectively regulate sodium and zinc in combination, resulting in reduced performance (i.e. lower survival, longer development time, smaller adult body size and slower growth rate). In contrast, the co-limitation hypothesis is based on the idea that metabolic pathways consist of complex nutrient co-limitation cascades, in which increased availability of one nutrient increases demand for another (Kaspari and Powers, 2016; Kaspari *et al.*, 2017; Browning *et al.*, 2017). This hypothesis predicts that if sodium and zinc are co-limiting in metabolic pathways, combined exposure to these micronutrients will positively impact monarch performance (i.e. increased survival, shorter development time, larger body size, faster growth rate). Given the involvement of sodium in neural and muscle development (Hodgkin, 1951), our second aim was to test for dietary micronutrient effects on butterfly eye area (a proxy for butterfly neural development; Ali, 1974) and thorax mass (a proxy for butterfly flight muscle development; Srygley and Chai, 1990). We hypothesized that exposure to elevated sodium levels would have positive effects on neural and thorax development (Snell-Rood *et al.*, 2014). Our third aim was to test for the effects of larval dietary sodium and zinc on the concentrations of both micronutrients in adult butterflies. Given that sodium and zinc are limited dietary micronutrients (Kaspari and Powers, 2016), we predicted that monarchs would have higher micronutrient concentrations in their tissues than the levels present in their food. Overall, because our sodium and zinc concentrations were either matched to the highest levels observed along roadsides (Na) or significantly above the maximum values (Zn), we expected relatively more support for the stress hypothesis.

## Materials and methods

### Origin of butterflies

Our rearing experiment used second-generation monarchs that originated from *D. plexippus* larvae captured on the University of Minnesota Saint Paul campus over a 1-week period in June 2020. We captured caterpillars at various stages of development, but all ranged from 2nd to 5th instar. All captured caterpillars were reared outdoors in Bug Dorm enclosures (61 × 61 × 61 cm) on common milkweed (*A. syriaca*) collected from gardens on campus. At adult emergence (i.e. eclosion), we maintained all butterflies outdoors in Bug Dorm enclosures and provided them with *ad libitum* access to sponges soaked with 10% honey water. We provided fresh honey water daily. Each cage contained a single host plant (*A. syriaca*) provided in a water wick along with a moist towel to maintain humidity. Each day, we transferred host plants with eggs to separate enclosures to allow larvae to hatch prior to being assigned to experimental treatments. We collected all eggs used in the experiment during a period from 15 to 21 July 2020. Eggs used in the experiment were derived from 12 breeding females. Breeding females were housed in two separate cages, and each cage had an equal sex ratio throughout the duration of the mating and egg collection period. Development of monarchs reared in the experiment took place from mid-July to late August. Adult monarchs eclosed from 13 to 28 August and were thus of the migratory generation, cued by the declining photoperiod and outdoor day-night temperature swings (Goehring and Oberhauser, 2002).

### Larval rearing on zinc- and sodium-treated milkweed

Monarch larvae were reared on *A. syriaca* plants. To minimize possible spatial variation in milkweed quality or element content, we collected all plants used in the experiment from gardens on the University of Minnesota Saint Paul campus over a 2-week period in July 2020. We reared monarch larvae in a 2 × 2 factorial design consisting of two factors (zinc or sodium) with each factor consisting of two levels (control or elevated). Our elevated sodium and zinc treatments were designed to approximate those used in prior research. Specifically, we aimed for an elevated zinc treatment of 344–616 mg/kg, which is around the minimum concentration range previously shown to negatively impact monarch survival in a laboratory study where zinc was administered to monarchs using an artificial diet (Shephard *et al*., 2020). We aimed for an elevated sodium treatment of ~2000 mg/kg, which is a concentration previously detected in roadside milkweed that had stressful effects in monarchs (Snell-Rood *et al.*, 2014). To prepare the control sodium/elevated zinc treatment combination, we first added 2.09 ml of 1 M ZnCl_2_ solution to 1 l of deionized water. To prepare the elevated sodium/control zinc treatment combination, we added 2.1 g of NaCl to 0.74 l of deionized water. To prepare the elevated sodium/elevated zinc treatment combination, we first added 2.09 ml of 1 M ZnCl_2_ solution to 1 l of deionized water and then added 0.74 l of this solution to 2.1 g of NaCl. The control sodium/control zinc treatment combination consisted of only deionized water. To apply these treatment combinations to milkweed plants, we sprayed the front and back side of each leaf of the plant with a single ~1.6-ml spray of solution from a Rubbermaid 0.74-l spray bottle. All plants were sprayed with treatments immediately after they were collected from the field. Before plants were introduced to the caterpillars, they were left to sit for 2–3 hours or until the sprayed water dried. This spray method allows for accumulation of micronutrients on leaf surfaces, which is one of the major ways that roadside plants accumulate metals in the field (e.g. up to 50% of leaf metal content in roadside plants has been attributed to dust accumulation; Shakour and Nasralla, 1986). Although some sodium contamination in roadside milkweed leaves may derive from leaf surface accumulation of dust from salty roadside soil, most contamination probably occurs through root uptake of sodium from the soil given the growing season of milkweed relative to the timing of salt application.

Final leaf zinc and sodium concentrations were confirmed by ICP-AES on five dry leaf samples from each treatment. Leaf zinc concentrations were 29.52 mg/kg and 555.91 mg/kg by dry weight, for the control and elevated zinc treatments, respectively. Leaf sodium concentrations were 77.83 mg/kg and 2087.95 mg/kg for the control and elevated sodium treatments, respectively. The elevated zinc treatment exceeds levels of zinc found in milkweed along roadsides, and our elevated sodium treatment is within the upper end of the range of sodium commonly found in roadside milkweed (Snell-Rood *et al.*, 2014; Mitchell *et al.*, 2020).

We gently transferred monarch larvae with a paintbrush at the second instar stage (5 days after egg collection) to milkweed dosed with one of the four treatment combinations: control sodium/control zinc, elevated sodium/control zinc, control sodium/elevated zinc or elevated sodium/elevated zinc. For the duration of the experiment, larvae were reared on milkweed in outdoor Bug Dorm enclosures. To keep larval density low, we set up two Bug Dorm enclosures for each of the four treatment combinations with 25 larvae assigned to each enclosure. This produced a total of 50 larvae in each treatment combination. The Bug Dorm enclosures were interspersed spatially in a partially shaded outdoor area, secured to the ground with rocks. Larvae were left in the outdoor enclosures to pupate and eclose.

### Measurements of life history traits

We calculated development time as the time between the date larvae were transferred to each milkweed treatment and the day of eclosion. We quantified survival as whether an individual survived from larva to eclosion. At eclosion, each butterfly was sexed, labelled with a single identification number and immediately transferred to a freezer.

For all butterflies, we measured total wing area as a proxy for adult body size. Total wing area was defined as the area of one forewing and one hindwing. To take this measurement, we used forceps to carefully remove a single forewing and hindwing from each butterfly, laid them flat and photographed them. Wing area measurements were conducted using ImageJ (NIH). We calculated growth rate for each individual as forewing area divided by development time.

### Measurements of neural and muscle investment

We measured butterfly eye area (relative to wing size) as a proxy for neural development. We measured dry thorax mass (relative to wing size) as a proxy for muscle development as total thorax mass is closely related to flight muscle mass in butterflies (Srygley and Chai, 1990). Before measuring thorax mass, we removed the head, abdomen and wings from each thorax, and then thoraxes were dried at 70°C for 24 hours. Relative eye area is an appropriate measure for neural investment in butterflies given that ~75% of the butterfly brain is devoted to visual processing (Ali, 1974). In addition, relative eye size is positively related to brain size in vertebrates and invertebrates (Gronenberg and Hölldobler, 1999; Garamszegi *et al.*, 2002). To measure eye area, butterfly heads were first imaged laterally following Rutowski (2000), and the area of each eye was measured using ImageJ (NIH). Because we were interested in relative investment in sodium-related traits, rather than simply trait scaling with body size, we focused on eye area and thorax mass relative to wing size (see statistical analyses below).

### Measurements of butterfly sodium and zinc concentrations

ICP-MS was performed at the Quantitative Bio-element Imaging Centre at Northwestern University to measure sodium and zinc concentrations in adult monarch butterfly abdomens (6 butterflies per treatment). We focused on the abdomen because it the largest body segment by mass of the adult butterfly. Prior to all analyses, abdomens were dried in a drying oven at 70°C for 24 hours. Monarch abdomens were then digested in concentrated trace nitric acid (>69%, Thermo Fisher Scientific, Waltham, MA, USA) at room temperature for 16 hours. Analyses were performed on a computer-controlled (QTERGA software) Thermo iCapQ ICP-MS (Thermo Fisher Scientific, Waltham, MA, USA).

### Statistical analyses

All statistical analyses were conducted in R Studio version 3.5.1. Survival to eclosion was quantified using a generalized linear mixed-effects model with a binomial distribution and a logit link function. This model included sodium treatment, zinc treatment and the interaction between sodium and zinc treatment as fixed effects and experimental replicate (cage) as a random effect.

Development time, total wing area, growth rate, eye area, thorax mass, adult abdomen sodium concentration and adult abdomen zinc concentration were analysed using linear mixed-effects models in the lme4 package (Bates *et al.*, 2015). For each model, sodium treatment, zinc treatment, sex and the interaction between sodium and zinc treatment were included as fixed effects and cage was included as a random effect. We assessed model fit by inspection of residuals versus fitted values. Given that body size is often correlated with the size of morphological traits (McCoy *et al.,* 2006), we tested for correlations between total wing area (our proxy for body size) and both eye area and thorax mass across all adult monarchs. Total wing area was positively correlated with both eye area (*r* = 0.48, *P* < 0.001) and thorax mass (*r* = 0.47, *P* < 0.001), so we included total wing area as a fixed effect in our mixed-effects models for both of these traits to control for the effect of body size. Given that our main hypotheses are centred on testing the interaction between sodium and zinc exposure (see Introduction), we report all model results including the sodium x zinc interactive term in the main text (Table 1, 2). However, to help with interpretation of the main effects, we also report all model results with interaction terms removed in Table S1 (survival) and Table S2 (other life history and morphological traits).

**Table 1:** Linear mixed-effects models for effects of sodium (Na), zinc (Zn) and the interaction between sodium and zinc (Na x Zn), on life history traits, eye area, thorax mass and adult abdomen micronutrient concentrations in the monarch butterfly (*D. plexippus*)

Random effects			Fixed effects				
	Variance	*SD*		Estimate	*SE*	*t*	*P*
A. Development time, *N* = 182							
Replicate	0.092	0.320	(Intercept)	28.10	0.31	91.81	<0.001
			Elevated Na	1.46	0.42	3.47	0.024
			Elevated Zn	0.88	0.41	2.13	0.10
			Elevated Na x Elevated Zn	−0.35	0.59	−0.59	0.59
			Sex (male)	0.21	0.21	1.00	0.32
B. Total wing area, *N* = 175							
Replicate	1.69	1.30	(Intercept)	18.09	0.95	19.13	<0.001
			Elevated Na	−0.24	1.33	−0.18	0.86
			Elevated Zn	0.11	1.33	0.082	0.94
			Elevated Na x Elevated Zn	−0.12	1.88	−0.064	0.95
			Sex (male)	0.80	0.21	3.81	<0.001
C. Growth rate, *N* = 175							
Replicate	0.0023	0.048	(Intercept)	0.64	0.035	18.26	<0.001
			Elevated Na	−0.041	0.050	−0.83	0.45
			Elevated Zn	−0.014	0.050	−0.30	0.78
			Elevated Na x Elevated Zn	0.0043	0.070	0.060	0.95
			Sex (male)	0.023	0.0087	2.69	0.008
D. Eye area, *N* = 71							
Replicate	0.00	0.000	(Intercept)	1.67	0.38	4.30	<0.001
			Elevated Na	0.16	0.095	1.70	0.094
			Elevated Zn	0.067	0.090	0.75	0.46
			Elevated Na x Elevated Zn	−0.12	0.13	−0.90	0.37
			Total wing area	0.10	0.020	4.79	<0.001
			Sex (male)	0.26	0.065	3.98	<0.001
E. Thorax mass, *N* = 75							
Replicate	<0.001	0.0016	(Intercept)	0.011	0.021	0.54	0.59
			Elevated Na	−0.0081	0.0059	−1.38	0.24
			Elevated Zn	−0.00095	0.0055	−0.17	0.87
			Elevated Na x Elevated Zn	−0.0020	0.0081	−0.25	0.81
			Total wing area	0.0047	0.0011	4.12	<0.001
			Sex (male)	0.00031	0.0040	0.079	0.94
F. Adult abdomen Na concentration, *N* = 24							
Replicate	0.00	0.00	(Intercept)	153.58	80.90	1.90	0.073
			Elevated Na	497.80	102.32	4.86	<0.001
			Elevated Zn	−9.50	102.32	0.093	0.93
			Elevated Na x Elevated Zn	193.87	144.71	1.34	0.20
			Sex (male)	121.24	72.36	1.68	0.11
G. Na concentration ratio (adult abdomen:larval foodplant), *N* = 24							
Replicate	0.63	0.79	(Intercept)	3.70	0.69	5.35	0.004
			Elevated Na	−3.22	0.94	−3.42	0.03
			Elevated Zn	−1.38	0.94	−1.47	0.22
			Elevated Na x Elevated Zn	−1.54	1.33	1.16	0.31
			Sex (male)	−0.33	0.37	−0.88	0.39
H. Adult abdomen Zn concentration, *N* = 24							
	0.00	0.00	(Intercept)	135.00	86.22	1.57	0.13
			Elevated Na	19.74	109.06	0.18	0.85
			Elevated Zn	768.03	109.06	7.04	<0.001
			Elevated Na x Elevated Zn	−249.82	154.24	−1.62	0.12
			Sex (male)	−57.52	77.12	−0.75	0.46
I. Zn concentration ratio (adult abdomen:larval foodplant), *N* = 24							
	0.00	0.00	(Intercept)	3.78	0.29	13.14	<0.001
			Elevated Na	0.59	0.36	1.61	0.12
			Elevated Zn	−2.53	0.36	−6.95	<0.001
			Elevated Na x Zn	0.31	0.51	0.60	0.55
			Sex (male)	−0.28	0.26	1.07	0.30

Model results with interaction terms removed are presented in Table S2.

We were also interested in understanding the degree to which monarchs concentrated sodium and zinc relative to the concentrations of these elements in their larval food sources. To do so, we calculated the ratio between the concentration of sodium or zinc present in each individual’s abdomen and the average concentration of either element in the larval food source. This is similar to methods used to measure bioconcentration factor in aquatic toxicology and plant biology (Gobas and Morrison, 2000). We used one-sample *t*-tests to determine whether average observed concentration ratios of sodium or zinc in each dietary treatment (control or elevated) were significantly different than 1. A concentration ratio over 1 indicates monarchs has a higher concentration of a micronutrient than the diet, a ratio of 1 indicates the monarch and diet concentrations are equivalent and a ratio less than one indicates the monarch has a lower concentration of a micronutrient than the diet.

## Results

### Effects of dietary sodium and zinc on monarch performance and development

**Figure 1:**
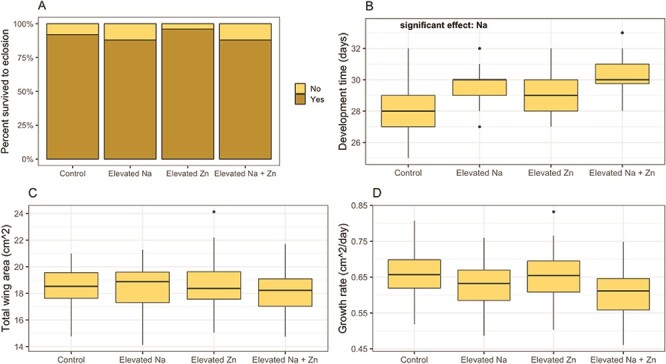
Effects of larval exposure to dietary sodium and zinc on survival and life history traits of monarch butterflies (*D. plexippus*). Each graph shows outcomes across the four treatment combinations used in our 2 × 2 factorial experiment (control, control sodium/control zinc; elevated Na, elevated sodium/control zinc, control sodium/elevated zinc; elevated Na + Zn, elevated sodium/elevated zinc). Survival (A) was quantified as the percentage of larvae in each treatment reaching adult eclosion. Development time (B) was measured as total number of days between the date larvae were transferred to each experimental treatment and the date of adult emergence. Total wing area (C) of adult butterflies was measured as the total area of one forewing and one hindwing. Growth rate (D) was calculated as total wing area divided by development time. Boxplot details: middle line shows median, box lengths show interquartile range, whiskers show upper and lower quartiles and points indicate outliers.

There were no significant effects of sodium [Estimate (SE) = −0.450 (0.679), *z* = −0.663, *P* = 0.51], zinc [Estimate (SE) = 0.736 (0.890), z = 0.826, *P* = 0.41] or sodium-by-zinc interaction [Estimate (SE) = −0.736 (1.082), z = −0.680, *P* = 0.50] on the probability of monarch survival from larva to adult eclosion (50 caterpillars reared in each of the four treatment combinations; Fig. 1A). However, there was a significant effect of sodium on development time (Table 1A), with individuals in the elevated sodium treatment taking ~1.3 days longer to develop (Fig. 1B). There were no significant effects of sodium, zinc or sodium-by-zinc interaction on total wing area (Table 1B; Fig. 1C) or growth rate (Table 1C; Fig. 1D).

**Figure 2:**
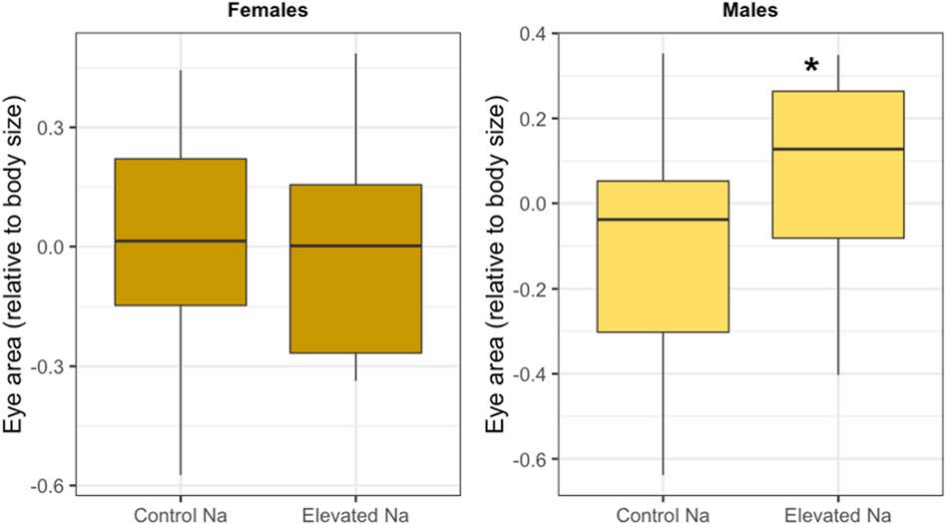
Effects of larval dietary exposure to sodium on eye area (relative to body size) in male and female monarch butterflies (*D. plexippus*). Eye area values are presented using the residuals from a regression analysis between total forewing area (proxy for adult body size) and average eye area across individual butterflies. Boxplot details: middle line shows median, box lengths show interquartile range and whiskers show upper and lower quartiles.

There were no significant effects of sodium, zinc or sodium-by-zinc interaction on monarch relative eye area (Table 1D) or relative thorax mass (Table 1E). Given that males developed significantly larger eyes than females (Table 1D), we also analysed effects of the micronutrient treatments on the sexes separately. Exposure to elevated dietary sodium significantly increased relative eye area in males but not females (Table 2, Fig. 2).

### Effects of dietary sodium and zinc on element concentrations in adult monarchs

**Figure 3:**
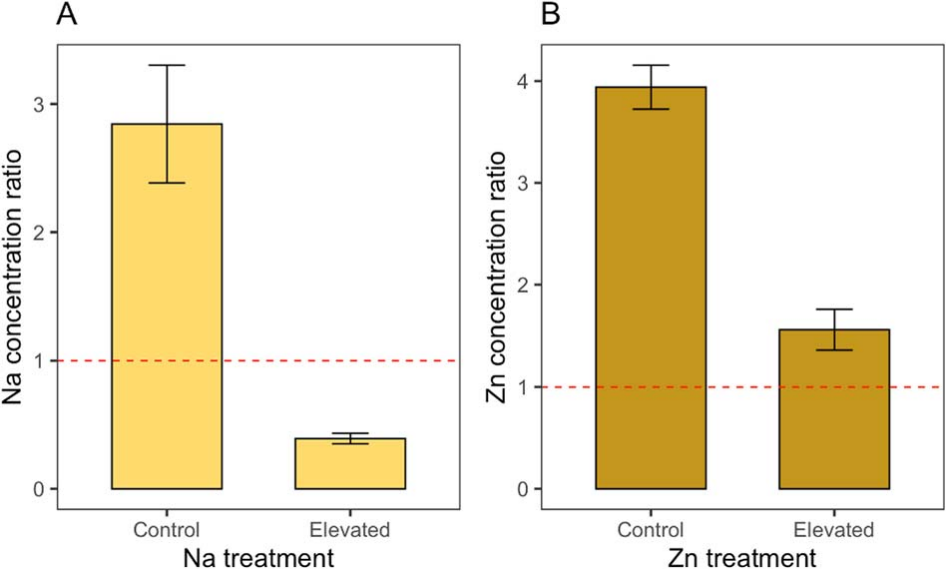
Concentration ratios of sodium (A) and zinc (B) between adult monarch abdomen and larval milkweed food source for monarchs reared on control milkweed or micronutrient-treated milkweed. Concentration ratios greater than 1 indicate that monarchs are concentrating micronutrients at levels higher than those present in their larval food sources. Bar plot details: height of the bar indicates the mean and bars indicate standard error.

Monarchs developing on sodium-treated milkweed as larvae had 3.8 times higher concentrations of sodium in their abdomens than monarchs feeding on control milkweeds (Table 1F). However, sodium concentration ratios between adult monarch abdomen and larval food plant were much lower for monarchs developing on sodium-treated milkweed than for monarchs developing on control milkweed (Table 1G; Fig. 3A). Specifically, monarchs developing on control plants had mean abdomen sodium concentrations of 209.45 mg/kg, which was 2.8 times higher than the host plants they were feeding on (*t* = 4.86, *P* = 0.002; Fig. 3A). However, those developing on sodium-treated plants had mean abdomen sodium concentrations of 804.19 mg/kg, which was 2.6 times lower than the host plants they were feeding on (*t* = 14.89, *P* < 0.001; Fig. 3A).

Monarchs developing on zinc-treated milkweed as larvae had 6.5 times higher zinc concentrations in their abdomens than monarchs feeding on control milkweeds (Table 1H). However, zinc concentration ratios between adult monarch abdomen and larval food plant were lower for monarchs developing on zinc-treated milkweed than for monarchs developing on control milkweed (Table 1I; Fig. 3B). Specifically, monarchs developing on control plants had mean abdomen zinc concentrations of 116.11 mg/kg, which was 3.9 times higher than the host plants they were feeding on (*t* = 13.61, *P* < 0.001; Fig. 3B). Those developing on zinc-treated plants had mean abdomen zinc concentrations of 759.22 mg/kg, which was 1.6 times higher than the host plants they were feeding on (*t* = 2.80, *P* = 0.02; Fig. 3B).

## Discussion

Human activities are altering the abundance and distribution of micronutrients in natural ecosystems, which may have significant effects on organismal development and fitness (Beanland *et al.*, 2003; Dehghani-Yakhdani *et al.*, 2019; Kaspari, 2020; Welti *et al.*, 2020). In the monarch butterfly, we tested the combined effects of dietary exposure to sodium and zinc, two of the most prominent human-released micronutrients contaminating monarch diet in roadside habitats (Mitchell *et al.*, 2020). Overall, we found no support for our initial hypotheses that combined sodium and zinc exposure would be stressful (i.e. negatively impact monarch performance) or co-limiting (i.e. positively impact monarch performance). Rather, monarch survival was not impacted by elevated sodium or zinc either individually or in combination (Fig. 1A). Additionally, while sodium exposure prolonged larval development (Fig. 1B), we found no effects of sodium, zinc or a combination of the two on growth rate or adult body size (Fig. 1C, D). Our results suggest that despite somewhat stressful effects of elevated sodium on monarch development, elevated sodium and zinc in combination are not negatively impacting monarch survival at levels typically observed in roadside plants (Mitchell *et al.*, 2020). This supports the positive potential of roadside habitats for monarch butterfly conservation (Kasten *et al.*, 2016).

**Table 2:** Linear mixed-effects models for effects of sodium (Na), zinc (Zn) and the interaction between sodium and zinc (Na x Zn), on eye area in male and female monarch butterflies (*D. plexippus*)

Random effects			Fixed effects				
	Variance	*SD*		Estimate	*SE*	*t*	*P*
Male eye area (log), *N* = 41							
Replicate	0.00	0.00	(Intercept)	1.59	0.58	2.75	0.009
			Elevated Na	0.26	0.12	2.11	0.042
			Elevated Zn	0.048	0.12	0.39	0.70
			Elevated Na x Elevated Zn	−0.14	0.16	−0.88	0.38
			Total wing area	0.12	0.029	3.99	<0.001
Female eye area (log), *N* = 30							
Replicate	0.00	0.00	(Intercept)	1.99	0.60	3.30	0.0029
			Elevated Na	0.029	0.17	0.17	0.86
			Elevated Zn	0.15	0.15	1.04	0.31
			Elevated Na x Elevated Zn	−0.096	0.23	−0.42	0.67
			Total wing area	0.082	0.034	2.44	0.022

Previous research suggests that the combined effects of chemical pollutants or stressors on organismal performance can often be far more detrimental than the effects of single stressors alone; yet, the vast majority of this work has focused on artificially produced chemicals (e.g. pesticides) or their interactions with naturally occurring stressors such as predation risk, food limitation or parasitism (e.g. Relyea and Mills, 2001; Sih *et al*., 2004; Crain *et al.*, 2008; Goulson *et al.*, 2015; Piggott *et al.*, 2015). Responses to micronutrients might play out differently because unlike pesticides or non-essential heavy metals, micronutrients are beneficial at low concentrations and organisms are therefore likely better equipped to effectively mitigate their potentially toxic effects through regulatory mechanisms such as sequestration or excretion (e.g. Bednarska *et al.*, 2015). For instance, insects can excrete excessive sodium through regulatory adjustment of the Malpighian tubules (O’Donnell, 2009), and excretion of sodium through the frass has been shown for grasshoppers feeding on sodium-elevated diets (Peterson *et al*., 2021). Sequestration or excretion of excess metals has been demonstrated as physiological mechanisms of heavy metal tolerance in both invertebrates and vertebrates. For example, populations of the soil invertebrate *Orchesella cincta* adapted to highly contaminated soils display an enhanced capacity to bind and excrete ingested metals (van Straalen *et al.*, 1987; Posthuma *et al.*, 1992), and this has been linked to evolutionary changes in metal-binding metallothionein pathways (Janssens *et al.*, 2009). High metal concentrations have also been found in melanized moults of birds and snakes (Chatelain *et al.*, 2014; Giraudeau *et al.*, 2015; Goiran *et al.*, 2017), suggesting that melanin pigments may aid in metal sequestration and excretion in vertebrates.

Our results suggest that monarch robustness to roadside micronutrients may in part be attributed to effective physiological regulation of internal zinc and sodium concentrations. For instance, compared to monarchs reared on control milkweed, we found that monarchs reared on sodium- or zinc-treated milkweed had much lower concentration ratios of each element between adult abdomen and larval food source (Table 1G, I; Fig. 3). This suggests that excretion of excess micronutrients during the larval stage probably played a significant role in monarch tolerance to sodium and zinc. However, monarchs reared on zinc-treated milkweed had zinc concentrations that were still 1.6 times higher than the zinc concentration of their food (Fig. 3B). This suggests that in addition to excreting zinc, monarchs may have been regulating dietary zinc in part through mechanisms of metal storage or sequestration. In animals, zinc is sequestered by metallothionein proteins, which enable zinc storage within the cell (Maret, 2000). Mechanisms of accumulation and sequestration have been shown to play a key role in zinc detoxification, including in butterflies and moths (Shu *et al.*, 2012; Jin *et al.*, 2020).

In contrast to the pattern observed for zinc concentration ratios, monarchs reared on sodium-treated milkweed had sodium concentrations that were 2.6 times lower than the sodium concentration of their food (Fig. 3A). This suggests that monarchs relied heavily on excretion to regulate internal levels of sodium. Unlike zinc, excess sodium cannot be stored in a stable form and must be excreted to avoid toxicity (NRC, 2005). Sodium regulation is extremely metabolically costly in animals (Frausto da Silva and Williams 2001), and we found that monarch caterpillars reared on sodium-treated milkweed took 1.3 days longer to reach adulthood (Table 1A; Fig. 1B). Therefore, while roadside sodium did not reduce monarch survival, it is possible that prolonged larval development time could be a cost of regulating internal sodium levels via excretion.

Consistent with our hypothesis that anthropogenic increases in sodium would positively affect neural development, we found that monarch larvae feeding on sodium-treated milkweed tended to develop larger eyes (Fig. 2). This result is consistent with previous work showing that anthropogenic increases in sodium positively affect butterfly neural development (Snell-Rood *et al.*, 2014). However, while Snell-Rood *et al*. (2014) found that larval dietary sodium enrichment increased neural development only in female monarchs and cabbage white butterflies (*Pieris rapae*), here we found that the effect was significant only in males (Table 2; Fig. 2). Although morphological responses to developmental dietary variation often differ between male and female butterflies (Merckx and Van Dyck, 2006; Pellegroms *et al.*, 2009), it remains uncertain why the pattern observed here was opposite to that observed by Snell-Rood *et al.* (2014). One possibility could be that dietary sodium enrichment may affect sex-specific neural development differently depending on whether or not monarchs are in reproductive diapause. For instance, in the study of reproductive monarchs by Snell-Rood *et al.* (2014), it is possible that females feeding on sodium-enriched milkweed may have invested more than males in neural production as larger eyes could assist females in searching locally for host plants for oviposition. However, given that our study was performed on migratory monarchs in reproductive diapause, increased investment in visual processing may be of little benefit to females. Alternatively, migratory females may already be at their physiological limit for eye size.

Sodium is a limiting micronutrient in animals (Kaspari and Powers 2016), and it is possible that with greater access to dietary sodium, monarchs reared on sodium-treated milkweed were able to invest more in neural development. Interestingly, we found that monarchs developing on sodium-treated milkweed also had prolonged development time (Table 1A; Fig. 1B). While prolonged development time could be a cost of sodium excretion (see above), it is also possible that this cost could arise as a consequence of increased investment in neural tissue. Delayed age at reproductive maturity is the most frequently hypothesized cost associated with investment in neural tissue (Laughlin *et al.*, 1998) and cognition more generally (Mayr, 1974; Dukas, 1998). As neural tissue is extremely metabolically expensive (Laughlin *et al.*, 1998), investing in a larger brain is expected to require energetic resources that might otherwise be allocated to the development of other adult body tissue. It is therefore possible that while increased sodium availability may fulfil the requirement for one limited resource needed to construct additional neural tissue, there may be other significant time or energy costs associated with the construction of this neural tissue that might delay maturation. In this regard, anthropogenic increases in sodium may result in novel limiting nutrients or other energetic resources that reveal new tradeoffs (see Snell-Rood *et al.*, 2015). Future work may therefore consider how other limiting nutritional or energetic resources (e.g. nitrogen availability) may interact with sodium availability to influence developmental costs of increased neural investment.

Despite positive effects of sodium on monarch neural development, we found no evidence that sodium positively affected flight muscle development (i.e. thorax mass; Table 1E). This is inconsistent with previous results showing that anthropogenic increases in sodium positively impacted butterfly flight muscle development (as measured by thorax protein content), particularly in males (Snell-Rood *et al.*, 2014). Although the elevated sodium treatment in our study (mean, 2088 mg/kg) is comparable to sodium levels in roadside-collected milkweed previously shown to enhance monarch muscle development (2065 mg/kg in Snell-Rood *et al.*, 2014), we speculate that this difference could again be attributed to the fact that the previous study used reproductive monarchs while our study used monarchs in reproductive diapause. Given that monarchs in reproductive diapause must fly 3000 miles across North America, it is possible that they already prioritize investment in flight muscle and would therefore not benefit from increased access to dietary sodium.

Our results add to a growing body of evidence supporting the positive conservation potential of roadside habitat for insect pollinators (Kasten *et al.*, 2016; Phillips *et al.*, 2019, 2020; Mitchell *et al.*, 2020; Shephard *et al*., in review). For instance, even though previous research has shown that there is a signature of zinc contamination across a range of roadside plant species used by insect pollinators, including milkweed, zinc levels do not reach levels that are known to be toxic for insect pollinators (Mitchell *et al.*, 2020; Shephard *et al.*, in review), including monarchs (Shephard *et al.*, 2020). The current study adds to these previous findings by showing that even in the presence of elevated sodium, zinc exposure does not negatively impact monarch survival (Fig. 1A). While our elevated sodium treatment (2088 mg/kg) is within the range of sodium concentrations detected in milkweed leaf tissue surveyed from Minnesota roadsides (minimum, 21; maximum, 4826 mg/kg; mean, 556 mg/kg; Mitchell *et al.*, 2020), our elevated zinc treatment (556 mg/kg) is far above the zinc concentration range found in roadside milkweed (minimum, 17; maximum, 54 mg/kg; mean, 33 mg/kg; Mitchell *et al.*, 2020). Thus, it is unlikely that combined exposure to elevated zinc and sodium are significantly impacting monarch survival at levels present in most roadside milkweed.

Although our results suggest that levels of dietary sodium and zinc in roadside habitats are not harmful for monarchs, the tolerance thresholds for zinc and sodium observed in our study are inconsistent with previously observed tolerance thresholds in monarchs. For instance, while we previously found that monarch survival was negatively impacted by zinc concentrations as low as 344 mg/kg (Shephard *et al.*, 2020), the current study found that monarchs were robust to 556 mg/kg zinc. Similarly, while we found that monarchs were robust to our elevated sodium treatment of 2088 mg/kg, previous work has shown that monarch survival is negatively impacted by similar sodium concentrations (Snell-Rood *et al.*, 2014; Kobiela and Snell-Rood, 2019). For instance, Snell-Rood *et al.* (2014) found that survival was reduced by 18% for monarchs feeding on roadside collected milkweed containing a similar sodium concentration of ~2065 mg/kg. While we cannot determine why tolerance thresholds differed across studies, it is possible that such differences could be attributed to variation in experimental rearing conditions. For example, while the current study was conducted by rearing monarchs on milkweed under field-like conditions (i.e. in outdoor enclosures), both previous studies were conducted in the laboratory. In the Shephard *et al*. (2020) study, zinc was administered to monarch caterpillars in the laboratory using an artificial diet that was likely quite stressful in itself, as monarch survival was only 18% in the control artificial diet treatment with no added zinc. It is possible that rearing in climate chambers in cups was additionally stressful relative to more field realistic conditions in outdoor cages. While Snell-Rood *et al.* (2014) reared monarch caterpillars in the laboratory on milkweed with elevated sodium levels, they collected all milkweed directly from roadsides. Milkweed growing along roadsides likely takes up some of its sodium from the soil, and this could negatively impact plant functioning (Zhu, 2007) and perhaps reduce plant nutritional quality. In the current study, we collected all milkweed from community gardens and sprayed all micronutrients on outer leaf surfaces. It is therefore possible that the milkweed used in our study could have been of greater overall nutritional quality than roadside-collected milkweed, potentially allowing monarchs to better tolerate sodium. Altogether, these studies highlight that organismal responses to environmental pollutants or stressors can be highly context dependent. This is important to consider when interpreting results of ecotoxicology studies, which are often conducted under laboratory conditions but tend to make inferences about conservation outcomes in the field. We therefore suggest that paying close attention to subtle factors that vary across ecotoxicology studies (e.g. rearing conditions, diet quality, density, etc.) will likely improve inferences about the generality of experimental findings, allowing us to better predict conservation outcomes.

In summary, we show that monarch survival is not negatively impacted by roadside sodium and zinc in combination, but roadside sodium is likely influencing phenotypic development more than zinc. Our results suggest several directions for future research. First, while we found that butterfly survival was not impacted by combined exposure to elevated levels of micronutrients, it remains to be determined whether micronutrient exposure might become more stressful in the presence of other common pollutants such as agrochemicals, or natural stressors such as temperature stress or water stress. Second, while our study confirms previous evidence showing that larval dietary sodium enrichment increases neural investment in butterflies (Snell-Rood *et al.*, 2014), the behavioural or fitness consequences of this effect (if any) remain unclear. While previous work found no support for the hypothesis that foraging monarchs are drawn to sodium-enriched milkweed either as larvae or adults (Mitchell *et al.*, 2019), it is unknown whether enhanced neural development as a result of dietary sodium enrichment confers benefits in terms of learning ability or host plant searching efficiency. Additionally, while anthropogenic increases in sodium may enhance development of sodium-limited traits such as brain size, it remains unclear whether these potential benefits produce novel tradeoffs (Snell-Rood *et al.*, 2015). If so, understanding whether the magnitude of such tradeoffs is influenced by variation in the availability of co-limiting nutrients (e.g. nitrogen) presents a virtually unexplored component of how anthropogenic increases in nutrient availability may alter selective dynamics.

## Funding

This work was supported by the Minnesota Environment and Natural Resources Trust Fund as recommended by the Legislative-Citizen Commission on Minnesota Resources. Element analysis was performed at the Northwestern University Quantitative Bio-element Imaging Center generously supported by NASA Ames Research Center [grant number NNA04CC36G].

## Supplementary Material

coab061_Supplemental_Material
