# Supplementary material for: Monarch caterpillars are robust to combined exposure to the roadside micronutrients sodium and zinc
Source: Conserv Physiol. 2021 Aug 9;9(1):coab061. doi: 10.1093/conphys/coab061 (PMC8354372; doi:10.1093/conphys/coab061)
Supplement: coab061_Supplemental_Material [file coab061_supplemental_material.docx]

**Table S1.** Generalized linear model results for effects of sodium (Na) and zinc (Zn) on survival from larva to eclosion in the monarch butterfly (*D. plexippus*).

|  | **Estimate** | ***SE*** | ***z*** | ***p*** |
| --- | --- | --- | --- | --- |
| (Intercept) | 2.63 | 0.48 | 5.53 | **<0.001** |
| Elevated Na | -0.76 | 0.52 | -1.46 | 0.14 |
| Elevated Zn | 0.25 | 0.50 | 0.50 | 0.62 |

**Table S2.** Linear mixed effects models for effects of sodium (Na) and zinc (Zn) on life-history traits, eye area, thorax mass, and adult abdomen micronutrient concentrations in the monarch butterfly (*Danaus plexippus*).

| **Random effects** | | | **Fixed effects** | | | | |
| --- | --- | --- | --- | --- | --- | --- | --- |
|  | **Variance** | ***SD*** |  | **Estimate** | ***SE*** | ***t*** | **p** |
| A. Development time; N = 182 | | | | | | | |
| replicate | 0.067 | 0.260 | (Intercept) | 28.18 | 0.25 | 110.13 | **<0.001** |
|  |  |  | Elevated Na | 1.29 | 0.27 | 4.68 | **0.0053** |
|  |  |  | Elevated Zn | 0.71 | 0.27 | 2.60 | **0.049** |
|  |  |  | Sex (male) | 0.22 | 0.21 | 1.09 | 0.30 |
| B. Total wing area; N = 175 | | | | | | | |
| replicate | 1.34 | 1.16 | (Intercept) | 18.12 | 0.74 | 24.60 | **<0.001** |
|  |  |  | Elevated Na | -0.30 | 0.84 | -0.36 | 0.74 |
|  |  |  | Elevated Zn | 0.049 | 0.84 | 0.058 | 0.96 |
|  |  |  | Sex (male) | 0.80 | 0.21 | 3.81 | **<0.001** |
| C. Growth rate; N = 175 | | | | | | | |
| replicate | 0.0018 | 0.043 | (Intercept) | 0.64 | 0.027 | 23.39 | **<0.001** |
|  |  |  | Elevated Na | -0.039 | 0.031 | -1.25 | 0.27 |
|  |  |  | Elevated Zn | -0.013 | 0.031 | -0.40 | 0.70 |
|  |  |  | Sex (male) | 0.023 | 0.0087 | 2.69 | **0.008** |
| D. Eye area; N = 71 | | | | | | | |
| replicate | 0.00 | 0.00 | (Intercept) | 1.69 | 0.39 | 4.35 | **<0.001** |
|  |  |  | Elevated Na | 0.10 | 0.064 | 1.52 | 0.13 |
|  |  |  | Elevated Zn | 0.010 | 0.064 | 0.16 | 0.87 |
|  |  |  | Total wing area | 0.10 | 0.021 | 4.82 | **<0.001** |
|  |  |  | Sex (male) | 0.27 | 0.065 | 4.11 | **<0.001** |
| E. Thorax mass; N = 75 | | | | | | | |
| replicate | 0.00 | 0.00 | (Intercept) | 0.011 | 0.021 | 0.57 | 0.57 |
|  |  |  | Elevated Na | -0.0093 | 0.0039 | -2.39 | **0.019** |
|  |  |  | Elevated Zn | -0.0018 | 0.0038 | -0.48 | 0.63 |
|  |  |  | Total wing area | 0.0047 | 0.0011 | 4.22 | **<0.001** |
|  |  |  | Sex (male) | 0.00045 | 0.0040 | 0.12 | 0.91 |
| F. Adult abdomen Na concentration; N = 24 | | | |  |  |  |  |
| replicate | 0.00 | 0.00 | (Intercept) | 105.11 | 73.78 | 1.42 | 0.17 |
|  |  |  | Elevated Na | 594.74 | 73.78 | 4.06 | **<0.001** |
|  |  |  | Elevated Zn | 87.44 | 73.78 | 1.18 | 0.25 |
|  |  |  | Sex (male) | 121.24 | 73.78 | 1.64 | 0.11 |
| G. Na concentration ratio (adult abdomen : larval foodplant); N = 24 | | | | | | | |
| replicate | 0.69 | 0.83 | (Intercept) | 3.31 | 0.62 | 5.31 | **0.002** |
|  |  |  | Elevated Na | -2.45 | 0.69 | -3.56 | **0.02** |
|  |  |  | Elevated Zn | -0.61 | 0.69 | -0.89 | 0.41 |
|  |  |  | Sex (male) | -0.33 | 0.37 | -0.89 | 0.39 |
| H. Adult abdomen Zn concentration; N = 24 | | | | | | | |
|  | 1693 | 41.15 | (Intercept) | 197.44 | 82.94 | 2.38 | **0.045** |
|  |  |  | Elevated Na | -105.17 | 85.08 | -1.25 | 0.27 |
|  |  |  | Elevated Zn | 643.12 | 84.08 | 7.65 | **<0.001** |
|  |  |  | Sex (male) | -57.50 | 79.41 | -0.72 | 0.48 |
| I. Zn concentration ratio (adult abdomen : larval foodplant); N = 24 | | | | | | | |
|  | 0.00 | 0.00 | (Intercept) | 3.70 | 0.25 | 14.63 | **<0.001** |
|  |  |  | Elevated Na | 0.74 | 0.25 | 2.93 | **0.008** |
|  |  |  | Elevated Zn | -2.38 | 0.25 | -9.38 | **<0.001** |
|  |  |  | Sex (male) | -0.28 | 0.25 | 1.09 | 0.29 |
